# Cervical screening attendance and cervical cancer risk among women who have sex with women

**DOI:** 10.1177/0969141320987271

**Published:** 2021-01-21

**Authors:** Catherine L Saunders, Efthalia Massou, Jo Waller, Catherine Meads, Laura AV Marlow, Juliet A Usher-Smith

**Affiliations:** 1 Primary Care Unit, University of Cambridge, Cambridge, UK; 2 Cancer Prevention Group, King's College London, London, UK; 3 Faculty of Health, Education, Medicine and Social Care, Anglia Ruskin University, Cambridge, UK

**Keywords:** Bowel screening, breast screening, cervical intraepithelial neoplasia, cervical screening, sexual minority health, women who have sex with women

## Abstract

**Objectives:**

To describe cervical cancer screening participation among women who have sex exclusively with women (WSEW) and women who have sex with women and men (WSWM) compared with women who have sex exclusively with men (WSEM), and women who have never had sex and compare this with bowel (colorectal) and breast screening participation. To explore whether there is evidence of differential stage 3 cervical intraepithelial neoplasia (CIN3) or cervical cancer risk.

**Methods:**

We describe cervical, bowel and breast cancer screening uptake in age groups eligible for the national screening programmes, prevalent CIN3 and cervical cancer at baseline, and incident CIN3 and cervical cancer at five years follow-up, among 218,674 women in UK Biobank, a cohort of healthy volunteers from the UK.

**Results:**

Compared with WSEM, in adjusted analysis [odds ratio (95% confidence interval)], WSEW 0.10 (0.08–0.13), WSWM 0.73 (0.58–0.91), and women who have never had sex 0.02 (0.01–0.02) were less likely to report ever having attended cervical screening. There were no differences when considering bowel cancer screening uptake (*p* = 0.61). For breast cancer screening, attendance was lower among WSWM 0.79 (0.68 to 0.91) and women who have never had sex 0.47 (0.29–0.58), compared with WSEM. There were incident and prevalent cases of both CIN3 and cervical cancer among WSEW and WSWM. Compared with WSEM with a single male partner, among WSEW there was a twofold increase in CIN3 1.91 (1.01 to 3.59); among WSWM with only one male partner, this was 2.25 (1.19 to 4.24).

**Conclusions:**

These findings highlight the importance of improving uptake of cervical screening among all women who have sex with women and breast screening among WSWM and women who have never had sex.

## Introduction

Cervical cancer accounted for 2% of all incident cancer in women in the UK in 2017 and is the 14th most common cancer diagnosed; incidence is highest among 30–34 year olds.^1^ In 2010–2012, 3.2% of all women and 4.7% of all 25–34-year-old women reported at least one female sexual partner in the previous five years.^2^ It is uncertain whether cervical cancer risk is higher or lower among women who have sex with women (WSW) because of variation in human papillomavirus (HPV) and other risk factor prevalence, and also disparities in screening history.

HPV is the main cause of cervical cancer. Our previous research found that HPV-associated cancers were those where there was greatest variation in risk among sexual minority women and men; however, we also found that women who report lesbian or bisexual sexual orientation were neither over- nor under-represented among women with cervical cancer.^3^ A recent systematic review identified increased risk of cervical cancer among bisexual but not lesbian women.^4^ Higher lifetime number of male sexual partners is a risk factor for HPV infection and cervical cancer, and WSW are more likely to have had higher numbers of male sexual partners.^5,6^ Genital HPV transmission also occurs among women who have sex exclusively with women (WSEW).^7,8^ Sexual minority women are likely to be younger, from more socially deprived backgrounds, and to smoke, compared with heterosexual women, all of which are also risk factors for cervical cancer.^2,6,9–12^

Modelling studies have shown that cervical screening in the UK has been effective in reducing cervical cancer,^13–15^ and ensuring equitable access to and uptake of screening among lesbian and bisexual women is a policy priority in the UK.^16–18^ Research from the UK and the US has found no difference in,^19^ or lower,^8,20,21^ cervical screening uptake among sexual minority women. However, there is little large-scale population-based data on screening uptake for any of the UK cancer screening programmes: breast, bowel (colorectal) or cervical.^17^ There have been calls from both the UK and the US to improve data on cancer outcomes in sexual minority women and men.^22,23^ A key challenge is that there are limited population-based or research databases or cohort studies where sexual orientation is prospectively recorded.^24^ UK Biobank is a prospective cohort study of healthy volunteers from the UK. History of sex with both same and opposite sex partners was asked at recruitment, and this cohort is emerging as an important resource for sexual minority health research.

Cervical screening programmes aim to detect cervical intraepithelial neoplasia (CIN), a treatable precursor to cervical cancer. There are three grades of CIN, of which CIN2 and CIN3 are treated when detected either through screening or symptomatic presentation. CIN3 diagnoses are recorded by cancer registries in the UK, and these data have also been linked to the UK Biobank cohort.^25^

In this analysis of data from UK Biobank, we describe screening attendance, and incident and prevalent CIN3 and cervical cancer, among WSW and women who have sex with men (WSM) in order to answer the following questions:

Is participation in screening for cervical cancer lower among WSW compared with women who have sex exclusively with men (WSEM), and is this reflected in lower cancer screening rates for other cancers as well? Is there any evidence of higher or lower CIN3 or cervical cancer risk among WSW?

## Methods

### Data

UK Biobank is a large cohort study of healthy volunteers from the UK recruited at age 37–70 between 2006 and 2010.^26,27^ A total of 9.2 million postal invitations were sent to people living within 25 miles of the 22 study assessment centres, and 503,325 people consented and were recruited, including 273,349 women. Touchscreen computers were used to collect survey questionnaire responses. The data used in this study were accessed through UK Biobank (application number 42,861).

### Screening history

Questions about screening history were asked to cohort members of all ages. For cervical screening, cohort members were asked “Have you ever had a cervical smear test?” with response options “Yes”, “No”, “Do not know” and “Prefer not to answer”. A second question asked "How many years ago was your last cervical smear test?" with numerical responses possible, and three additional options “Less than a year ago”, “Do not know” and “Prefer not to answer”.

For bowel screening, cohort members were asked “Have you ever had a screening test for bowel (colorectal) cancer? (Please include tests for blood in the stool/faeces or a colonoscopy or a sigmoidoscopy)” with response options “Yes”, “No”, “Do not know” and “Prefer not to answer”.

For breast screening, the question was “Have you ever been for breast cancer screening (a mammogram)?” again with response options “Yes”, “No”, “Do not know” and “Prefer not to answer”.

### CIN and cervical cancer diagnoses

CIN3 was defined as a cancer registry recorded ICD10 diagnosis code of “D06” or an ICD9 cancer registry code of 2331. Cervical cancer diagnoses were defined with an ICD10 code of “C53” or an ICD9 code of 180,018,011,808 or 1809.

We included prevalent cases of CIN3 and cervical cancer at baseline assessment defined as any cancer registry recorded diagnosis with a first date of diagnosis before the baseline assessment date.

Incident CIN3 and cervical cancer diagnoses were estimated for the first five years of follow-up after baseline assessment, as all cohort members had at least five years of complete follow-up after this date. People with prevalent CIN3 or cervical cancer before baseline assessment were excluded from these analyses of incident cancer.

### Sexual history

We identified women who had never had sex, WSEW, WSEM and WSWM based on responses to sexual history questions about lifetime numbers of same sex and opposite sex partners (details in Appendix Figure 1, see online Supplemental Material).

### Cohort characteristics

We described age, ethnicity and smoking history based on responses to the baseline touchscreen survey. Socio-economic deprivation was calculated using the Townsend score, a small area measure of material deprivation based on postcode of residence. We categorised deprivation into five groups based on national quintile defining cutpoints;^28^ people living in the most deprived areas of the UK are under-represented in UK Biobank.^26^ We also describe the recruitment year to UK Biobank because the UK bowel screening programme was rolled out between 2006 and 2010,^29^ and so for bowel screening there will have been variable uptake across the study period.

### Analysis

After excluding missing data (Appendix Table 1) in our first analysis, we described the characteristics and cervical cancer risk factor profiles. Because of the differences in the age profiles of women in these four groups, we estimated further descriptive statistics stratified by age.

To compare participation in screening for cervical cancer among WSW compared with WSEM and compare the patterns of participation with those for bowel and breast cancer screening, in our second analysis, we compared uptake of cervical, bowel and breast cancer screening in each group of women. For cervical cancer screening, we included all women reporting a history of ever having attended screening and additionally described up-to-date screening history (under 50 and screened in the last three years or 50–64 and screened within the last five years). We restricted the analysis to women over 50 for breast screening and women over 60 for bowel screening in line with screening programme ages. We explored screening uptake in adjusted analysis using logistic regression, adjusting for age, year of baseline assessment, deprivation and ethnicity.

In order to explore higher or lower CIN3 or cervical cancer risk among WSEW, WSWM and WSEM, we described unadjusted variation in prevalent and incident CIN3 and cervical cancer among women in each group. In adjusted analysis, using logistic regression, we only explored prevalent CIN3 because of small numbers of outcomes among WSW for prevalent cervical cancer or incident CIN3 or cervical cancer. In this adjusted analysis, considering history of CIN3 at baseline assessment, we described variation between women with no history of sex with women or men, WSEW, WSEM and WSWM adjusted for age, deprivation and smoking history. Because these four groups of women were defined on the basis of lifetime sexual history, numbers of sexual partners could not be incorporated into these models. In order to describe the relationship between lifetime numbers of male sexual partners and CIN3, we stratified the cohort by history of ever having sex with women, and in this analysis explored the relationship with lifetime numbers of male sexual partners separately in the two groups (women who reported ever, or never, having had sex with a woman). We could not stratify by lifetime numbers of female partners alone because of low numbers in these groups.

All data processing and analyses were carried out using Stata 15.1.

## Results

After excluding women for whom sexual history (50,531, 22.7%), socio-demographic information (1,591, 0.7%) or screening history (2,553, 1.2%) was missing, we included 218,674 women in this analysis.

One per cent of women (2192) reported no history of sex with either women or men, 0.3% (684) sex exclusively with women (WSEW), 210,866 (96.4%) sex exclusively with men (WSEM) and 4932 (2.3%) sex with both men and women (WSWM) (Table 1).

**Table 1.** Sample characteristics.

	No history of sex with either women or menN (%)	Sex exclusively with women (WSEW) N (%)	Sex exclusively with men (WSEM) N (%)	Sex with both women and men (WSWM) N (%)
Total				
	2192	684	210,866	4932
Age at baseline assessment				
Under 45	270 (12.3)	155 (22.7)	21,550 (10.2)	1090 (22.1)
45–49	335 (15.3)	175 (25.6)	28,681 (13.6)	1157 (23.5)
50–54	348 (15.9)	124 (18.1)	33,777 (16.0)	1019 (20.7)
55–59	372 (17.0)	89 (13.0)	39,829 (18.9)	815 (16.5)
60–64	484 (22.1)	89 (13.0)	51,370 (24.4)	624 (12.7)
65 and over	383 (17.5)	52 (7.6)	35,659 (16.9)	227 (4.6)
Lifetime number of sexual partners				
0	2192 (100)			
1		177 (25.9)	71,329 (33.8)	
2 to 3		171 (25.0)	55,324 (26.2)	609 (12.3)
4 to 5		134 (19.6)	35,251 (16.7)	755 (15.3)
6 or more		202 (29.5)	48,962 (23.2)	3568 (72.3)
Lifetime number of same sex partners				
0	2192 (100)		210,866 (100)	
1		177 (25.9)		2480 (50.3)
2 to 3		171 (25.0)		1456 (29.5)
4 to 5		134 (19.6)		485 (9.8)
6 or more		202 (29.5)		511 (10.4)
Lifetime number of opposite sex partners				
0	2192 (100)	684 (100)		
1			71,329 (33.8)	600 (12.2)
2 to 3			55,324 (26.2)	921 (18.7)
4 to 5			35,251 (16.7)	710 (14.4)
6 or more			48,962 (23.2)	2701 (54.8)
Deprivation				
Least deprived	525 (24.0)	184 (26.9)	82,236 (39.0)	1055 (21.4)
2	422 (19.3)	139 (20.3)	45,259 (21.5)	786 (15.9)
3	413 (18.8)	113 (16.5)	31,233 (14.8)	894 (18.1)
4	373 (17)	105 (15.4)	27,018 (12.8)	1024 (20.8)
Most deprived	459 (20.9)	143 (20.9)	25,120 (11.9)	1173 (23.8)
Ethnicity				
White	2103 (95.9)	628 (91.8)	202,580 (96.1)	4702 (95.3)
Mixed	10 (0.5)	8 (1.2)	1006 (0.5)	72 (1.5)
Asian	33 (1.5)	17 (2.5)	2431 (1.2)	21 (0.4)
Black	24 (1.1)	12 (1.8)	2773 (1.3)	81 (1.6)
Other	22 (1.0)	19 (2.8)	2076 (1.0)	56 (1.1)
Smoking history				
Never	1898 (86.6)	375 (54.8)	127,883 (60.6)	1832 (37.1)
Former	225 (10.3)	211 (30.8)	65,607 (31.1)	2167 (43.9)
Current	69 (3.1)	98 (14.3)	17,376 (8.2)	933 (18.9)
Year of baseline assessment				
2006	24 (1.1)	5 (0.7)	1599 (0.8)	40 (0.8)
2007	339 (15.5)	83 (12.1)	20,737 (9.8)	527 (10.7)
2008	794 (36.2)	248 (36.3)	79,628 (37.8)	1644 (33.3)
2009	645 (29.4)	218 (31.9)	72,635 (34.4)	1730 (35.1)
2010	390 (17.8)	130 (19.0)	36,267 (17.2)	991 (20.1)

WSEW and WSEM reported similar lifetime numbers of sexual partners, with 29.5% and 23.2% reporting six or more; 72.3% of WSWM reported six or more lifetime partners. WSWM reported fewer female sexual partners than WSEW and more male sexual partners than WSEM.

Overall, 39.0% of WSEM, 24.0% of women with no history of sex, 26.9% WSEW and 21.4% WSWM lived in the least deprived 20% of areas of the UK, and smoking history was highest among WSEW and WSWM (Table 1). Lifetime numbers of all and same sex partners were higher among younger women, although fewer women at older ages had never had sex with women or men. CIN3 history was higher among younger women, at 3.6% in under 45 year olds compared with 0.9% in over 65 year olds. Cohort characteristics are presented in Appendix Tables 2 and 3 (see online Supplemental Material).

### Participation in screening

We found that 45.7% of women with no history of sex with either women or men reported never having attended cervical cancer screening, and 22.0% of under 50 year olds and 27.2% of 50 and over year olds in this group reported up-to-date screening history.

Compared with 1.3% of WSEM and 1.6% of WSWM, 10.5% of WSEW had never attended cervical screening (Table 2). Overall, a history of ever having attended cervical screening was slightly higher among younger than older women (Appendix Table 3).

**Table 2.** Screening history.

	No history of sex with either women or men N (%)	Sex exclusively with women (WSEW) N (%)	Sex exclusively with men (WSEM) N (%)	Sex with both women and men (WSWM) N (%)
Cervical screening				
Never screened	1001 (45.7)	72 (10.5)	2806 (1.3)	78 (1.6)
Ever screened	1191 (54.3)	612 (89.5)	208,060 (98.7)	4854 (98.4)
Cervical screening within the last three years (under 50)				
No	472 (78.0)	127 (38.5)	7760 (15.5)	407 (18.1)
Yes	133 (22.0)	203 (61.5)	42,471 (84.6)	1840 (81.9)
Cervical screening within the last five years (50–64)				
No	877 (72.8)	100 (33.1)	31,488 (25.2)	592 (24.1)
Yes	327 (27.2)	202 (66.9)	93,488 (74.8)	1866 (75.9)
Bowel screening (60+ only)				
Never screened	454 (52.4)	71 (50.4)	42,888 (49.3)	411 (48.3)
Ever screened	413 (47.6)	70 (49.6)	44,141 (50.7)	440 (51.7)
Breast screening (50+ only)				
Never screened	123 (7.8)	29 (8.2)	6273 (3.9)	218 (8.1)
Ever screened	1464 (92.2)	325 (91.8)	154,362 (96.1)	2467 (91.9)

Considering all screening history, in adjusted analysis [Odds ratio (OR) (95% confidence interval, CI)], WSEM were the most likely group to have ever had cervical screening (joint p-value <0.0001), and WSEW were 10 times less likely to have ever attended 0.10 (0.08 to 0.13). No difference was seen for ever having had bowel screening (*p* = 0.61), and for breast cancer screening, uptake was lower among WSWM 0.79 (0.86–0.91) and women who had never had sex 0.47 (0.29 to 0.58), Table 3. In further analysis, where we considered only women with an up-to-date cervical screening history, differences between WSEW and WSWM compared with WSEM attenuated somewhat (Table 3).

**Table 3.** Predictors of screening attendance.

	OR (95%CI)	p-value	OR (95%CI)	p-value
	Cervical screening (ever)		Cervical screening (up to date at baseline assessment, under 65 only)	
No history of sex with either women or men	0.02 (0.01 to 0.02)	<0.0001	0.09 (0.08 to 0.1)	<0.0001
Sex exclusively with women (WSEW)	0.10 (0.08 to 0.13)		0.42 (0.35 to 0.49)	
Sex exclusively with men (WSEM)	Reference		Reference	
Sex with both women and men (WSWM)	0.73 (0.58 to 0.91)		0.91 (0.84 to 0.97)	
	Bowel screening (over 60 only)			
No history of sex with either women or men	0.96 (0.83 to 1.11)	0.61		
Sex exclusively with women (WSEW)	0.87 (0.61 to 1.24)			
Sex exclusively with men (WSEM)	Reference			
Sex with both women and men (WSWM)	0.93 (0.81 to 1.08)			
	Breast screening (over 50 only)			
No history of sex with either women or men	0.47 (0.39 to 0.58)	<0.0001		
Sex exclusively with women (WSEW)	0.70 (0.47 to 1.05)			
Sex exclusively with men (WSEM)	Reference			
Sex with both women and men (WSWM)	0.79 (0.68 to 0.91)			

Note: Adjusted for age, ethnicity, year of baseline assessment, and deprivation.

### Incident and prevalent CIN3 and cervical cancer

There were 4386 women (2.0%) in the cohort who had a history of CIN3 at baseline assessment (prevalent CIN3) and 196 (0.1%) reports of CIN3 during the first five years of follow-up (incident CIN3). Overall, 731 (0.3%) women had a history of cervical cancer at baseline assessment with 46 incident cases during the first five years of follow-up; 231 cases (31.9%) of cervical cancer occurred among people with no history of CIN3. Women with all reported sexual histories (no sexual history, WSEW, WSEM and WSWM) were represented among both CIN and cervical cancer cases (Table 4).

**Table 4.** Incident and prevalent CIN3 and cervical cancer.

	All women	No history of sex with either women or men	Sex exclusively with women (WSEW)	Sex exclusively with men (WSEM)	Sex with both women and men (WSWM)
Prevalent CIN3 at baseline assessment		
No	214,288 (98.0)	2189 (99.9)	674 (98.5)	206,626 (98)	4799 (97.3)
Yes	4386 (2.0)	3 (0.1)	10 (1.5)	4240 (2)	133 (2.7)
Incident CIN3 within five years of follow up (n = 214,288)^a^		
No	214,092 (99.0)	2189 (100)	674 (100)	206,434 (99.9)	4795 (99.9)
Yes	196 (0.1)	0 (0)	0 (0)	192 (0.1)	4 (0.1)
Prevalent cervical cancer at baseline assessment					
No	217,943 (99.7)	2190 (99.9)	683 (99.9)	210,156 (99.7)	4914 (99.6)
Yes	731 (0.3)	2 (0.1)	1 (0.1)	710 (0.3)	18 (0.4)
Incident cervical cancer within 5 years of follow up (n = 217,943)^a^					
No	217,897 (100.0)	2189 (100)	683 (100)	210,112 (100)	4913 (100)
Yes	46 (0.0)	1 (0)	0 (0)	44 (0)	1 (0)

a The denominator for incident CIN3/cervical cancer excludes prevalent cases at baseline.

In multivariable analysis, women who had never had sex had a greater than 10-fold lower risk of a history of CIN3 compared with WSEM. Overall, after adjusting for age, deprivation and smoking history, WSEW and WSWM had lower odds of a recorded CIN3 history compared with WSEM, although the 95%CI cross 1 (Table 5). WSW with a history of no male partners or only one male partner had a twofold increase in CIN3 compared with WSEM with a history of only one male partner; OR (95%CI) 1.91 (1.01 to 3.59) for WSW with a history of no male partners, and 2.25 (1.19 to 4.24) for WSW with only one male partner (Table 5).

**Table 5.** Association between same and opposite sex sexual history and history of CIN3 at UK Biobank baseline assessment.

Model 1. Association between sexual history and history of prevalent CIN3			
	Unadjusted	Adjusted for age, deprivation	Adjusted for age, deprivation, smoking history
	OR (95%CI)	OR (95%CI)	OR (95%CI)
No history of sex with either women or men	0.07 (0.02 to 0.21)	0.06 (0.02 to 0.19)	0.08 (0.02 to 0.24)
Sex exclusively with women (WSEW)	0.72 (0.39 to 1.35)	0.56 (0.30 to 1.06)	0.53 (0.28 to 1.00)
Sex exclusively with men (WSEM)	Reference	Reference	Reference
Sex with both women and men (WSWM)	1.35 (1.13 to 1.61)	1.02 (0.85 to 1.21)	0.86 (0.72 to 1.03)
joint p-value	<0.0001	<0.0001	<0.0001
Model 2. Including lifetime numbers of male sexual partners, adjusted for age, deprivation, smoking history			
No history of sex with women (women who have never had sex and WSEM)		Women who have sex with women (WSEW and WSWM)	
Lifetime number of male sexual partners	OR (95%CI)	Lifetime number of male sexual partners	OR (95%CI)
0	0.23 (0.07 to 0.72)	0	1.91 (1.01 to 3.59)
1	reference	1	2.25 (1.19 to 4.24)
2 to 3	2.70 (2.39 to 3.04)	2 to 3	2.50 (1.55 to 4.04)
4 to 5	4.05 (3.59 to 4.57)	4 to 5	1.90 (1.04 to 3.48)
6 or more	5.37 (4.79 to 6.02)	6 or more	3.98 (3.15 to 5.03)

## Discussion

WSEW, WSWM and women who have never had sex are less likely than WSEM to report ever having attended cervical screening. There was no difference between the four groups when considering bowel cancer screening uptake. For breast screening, WSWM and women who have never had sex report lower screening uptake compared with WSEM. In linked cancer registry data from UK Biobank, there were incident and prevalent cases of both CIN3 and cervical cancer among WSEW and WSWM. In addition, WSEW were more likely to have a history of CIN3 than women who reported never having sex or than WSEM with only one sexual partner.

Our finding that lower uptake among WSEW is seen for cervical screening, but not for bowel or breast, suggests that it is likely to be due to issues specific to the cervical cancer screening process. This may relate to historic messaging which specifically discouraged cervical screening among lesbian and bisexual women in the UK^21^ (it was only in 2009 that the UK government began to proactively promote cervical screening uptake among lesbian and bisexual women^30^) or because opportunities for cervical screening are missed if they are routinely carried out during contraceptive review appointments, which lesbian and bisexual women may be less likely to attend.^31^ The practice of refusing contraception prescriptions unless cervical screening is completed may exacerbate these differences.^32^ Dislike of, or pain associated with, the use of a speculum during cervical screening may be a further reason for lower screening uptake among some WSEW, although most lesbian and bisexual women report vaginal penetration occasionally or often with female or male sexual partners.^33^

Challenges in accessing healthcare among lesbian, gay and bisexual women,^34^ or generally poorer experiences of primary or cancer care,^11,35,36^ remain important issues for the health of sexual minority women. As cervical screening is the only one of the three UK screening programmes carried out in primary care, these may be additional possible explanations for the specifically lower attendance for cervical screening.

Our findings that WSEW and WSWM are represented among women with a history of CIN3 or cervical cancer highlight the ongoing importance of screening among these groups. Although, on average, WSEW are about half as likely to have a history of CIN3 than WSEM, we find no statistical evidence of reduced risk when compared with all WSEM (95% confidence intervals include 1). This lower risk should therefore not be over-interpreted, however cautiously it may be explained by either lower screening attendance or lower risk of CIN3 associated with sex exclusively with women or a combination of both. Our finding that WSEW have a higher prevalence of CIN3 when compared only with women who have had a single male partner is an important indication that lesbian compared with heterosexual sex alone cannot be the only explanation for this lower risk, again highlighting the importance of screening among these groups. We additionally find no evidence to support the possibility that higher cervical cancer risk among lesbian and bisexual women is explained by higher numbers of male sexual partners in these groups.^4^

Cervical cancer screening is associated with a 60% reduction in cancer in women aged 40 and 80% at age 64.^14^ Three per cent of women treated for CIN3 would have had cancer by age 40 had the CIN3 not been treated.^13^ Interventions to encourage uptake of screening among sexual minority women should be particularly targeted at cervical cancer, and our analysis suggests that the current policy focus is warranted.^16,17^ Women who have never had sex are less likely to have ever had cervical screening, but given the known aetiology associated with HPV infection, cervical cancer risk is also likely to be substantially lower in this group; there is a less strong argument for targeting cervical screening among these women. However, our finding that mammography uptake is also lower, despite evidence of higher breast cancer risk associated with nulliparity,^37^ suggests this is a group to whom breast cancer screening uptake interventions could potentially be targeted. This analysis also highlights the importance of the collection or linkage of sexual orientation and sexual behaviour information for UK cancer registries to allow disparities in cancer outcomes to be monitored and reported.

The key strengths of this analysis are the large sample size and the cancer registry linked cancer outcomes data, combined with self-reported screening information and sexual history for women in the UK Biobank cohort. Public Health England have highlighted the lack of data available on screening uptake among the lesbian, gay, bisexual, transgender population,^17^ and this analysis goes part of the way towards addressing this evidential need.

There are limitations to this analysis. UK Biobank is not representative of the UK population^26^ and, specifically relevant to this analysis, our finding that cervical screening history is higher among younger compared with older women varies from the pattern seen in the UK as a whole.^38^ This is the strongest limitation to this work; to address this concern, we adjusted for age, deprivation and ethnicity in multivariable analysis. A second limitation is that we explored sexual history rather than sexual orientation, and although this may be appropriate for analyses exploring disparities in cancer outcomes associated with sexually transmitted HPV infection,^3^ it should be highlighted that most WSWM identify as heterosexual rather than bisexual or lesbian,^39^ and so caution is needed before using these results to describe disparities associated with sexual identity.

A third limitation is that we could not specifically explore screening uptake associated with national bowel and breast screening programmes because the measure of screening in UK Biobank could incorporate both diagnostic testing and asymptomatic screening. In this analysis, screening for cervical cancer is different from bowel and breast in the context of the questions asked; for bowel and breast screening, the investigations could be being done following symptomatic presentation, but cervical cancer smear tests cannot be carried out in primary care outside the context of the national screening programme.

Regarding CIN3 and cancer outcomes, there is the potential for survivor bias in this analysis, although the low mortality overall from CIN3 will mitigate this somewhat. Further, for analyses of CIN3, we were not able to adjust for screening history, as almost everyone with a history of CIN3 reported having attended screening at least once in their lifetime. We include incident and prevalent cervical cancer in this analysis in descriptive statistics, as these diagnoses represent cases of cancer not prevented by screening, although numbers were not high enough for a multivariable analysis.

Age, screening history, sexual history and CIN3 risk are all related. In addition to cohort effects in screening programme roll-out, it is possible also that completeness of cancer recording varies over time. The descriptive age and socio-demographic stratified supplementary analyses shed some more light on these relationships.

Despite these limitations, this analysis presents novel findings from a large cohort of women in the UK. For cervical screening, we find lower uptake among WSW (both WSEW and WSWM), a finding that is not mirrored in bowel screening uptake. We also show that WSEW and WSWM are represented among all women with CIN3 and cervical cancer diagnoses, and that WSEW have higher CIN3 prevalence than women who have had one or zero male partners across their lifetimes, confirming the risk of HPV transmission associated with lesbian sex. Together, these findings highlight the importance of policy interventions to improve uptake of cervical screening among women who have sex with women overall.

## Supplemental Material

sj-pdf-1-msc-10.1177_0969141320987271 - Supplemental material for Cervical screening attendance and cervical cancer risk among women who have sex with women Click here for additional data file. Supplemental material, sj-pdf-1-msc-10.1177_0969141320987271 for Cervical screening attendance and cervical cancer risk among women who have sex with women by Catherine L Saunders, Efthalia Massou, Jo Waller, Catherine Meads, Laura AV Marlow and Juliet A Usher-Smith in Journal of Medical Screening

sj-pdf-2-msc-10.1177_0969141320987271 - Supplemental material for Cervical screening attendance and cervical cancer risk among women who have sex with women Click here for additional data file. Supplemental material, sj-pdf-2-msc-10.1177_0969141320987271 for Cervical screening attendance and cervical cancer risk among women who have sex with women by Catherine L Saunders, Efthalia Massou, Jo Waller, Catherine Meads, Laura AV Marlow and Juliet A Usher-Smith in Journal of Medical Screening

## References

[1] Cancer Research UK. *Cervical cancer incidence statistics* 2020, www.cancerresearchuk.org/health-professional/cancer-statistics/statistics-by-cancer-type/cervical-cancer/incidence (accessed 31 December 2020).

[2] Mercer CH Tanton C Prah P, et al. Changes in sexual attitudes and lifestyles in Britain through the life course and over time: findings from the national surveys of sexual attitudes and lifestyles (natsal). Lancet 2013; 382: 1781–1794. 24286784 10.1016/S0140-6736(13)62035-8 PMC3899021

[3] Saunders CL Meads C Abel GA, et al. Between sexual orientation and overall and site-specific diagnosis of cancer: evidence from two national patient surveys in England. J Clin Oncol 2017. 35(32):3654–3661. 28945501 10.1200/JCO.2017.72.5465 PMC5855217

[4] Robinson K Galloway KY Bewley S, et al. Lesbian and bisexual women's gynaecological conditions: a systematic review and exploratory meta-analysis. BJOG 2017; 124: 381–392. 27862853 10.1111/1471-0528.14414 PMC5363366

[5] Hodson K Meads C Bewley S. Lesbian and bisexual women's likelihood of becoming pregnant: a systematic review and meta-analysis. BJOG 2017; 124: 393–402. 27981741 10.1111/1471-0528.14449 PMC5299536

[6] Mercer CH Bailey JV Johnson AM, et al. Women who report having sex with women: British national probability data on prevalence, sexual behaviors, and health outcomes. Am J Public Health 2007; 97: 1126–1133. 17463372 10.2105/AJPH.2006.086439 PMC1874216

[7] Marrazzo JM Stine K Koutsky LA. Genital human papillomavirus infection in women who have sex with women: a review. Am J Obstet Gynecol 2000; 183: 770–774. 10992207 10.1067/mob.2000.106681

[8] Marrazzo JM. Genital human papillomavirus infection in women who have sex with women: a concern for patients and providers. AIDS Patient Care STDS 2000; 14: 447–451. 10977974 10.1089/108729100416669

[9] Hagger-Johnson G Taibjee R Semlyen J, et al. Sexual orientation identity in relation to smoking history and alcohol use at age 18/19: cross-sectional associations from the longitudinal study of young people in England (LSYPE). BMJ Open 2013; 3: e002810. 10.1136/bmjopen-2013-002810 PMC3758978 23985386

[10] Cochran SD Mays VM Bowen D, et al. Cancer-related risk indicators and preventive screening behaviors among lesbians and bisexual women. Am J Public Health 2001; 91: 591–597. 11291371 10.2105/ajph.91.4.591 PMC1446636

[11] Elliott MN Kanouse DE Burkhart Q, et al. Sexual minorities in England have poorer health and worse health care experiences: a national survey. J Gen Intern Med 2015; 30: 9–16. 25190140 10.1007/s11606-014-2905-y PMC4284269

[12] Douglas E Waller J Duffy SW, et al. Socioeconomic inequalities in breast and cervical screening coverage in England: are we closing the gap? J Med Screen 2016; 23: 98–103. 26377810 10.1177/0969141315600192 PMC4855247

[13] Sasieni P Castanon A Parkin DM. How many cervical cancers are prevented by treatment of screen-detected disease in young women? Int J Cancer 2009; 124: 461–464. 18844220 10.1002/ijc.23922

[14] Sasieni P Castanon A Cuzick J. Effectiveness of cervical screening with age: population based case-control study of prospectively recorded data. BMJ 2009; 339: b2968. 19638651 10.1136/bmj.b2968 PMC2718082

[15] Landy R Pesola F Castanon A, et al. Impact of cervical screening on cervical cancer mortality: estimation using stage-specific results from a nested case-control study. Br J Cancer 2016; 115: 1140–1146. 27632376 10.1038/bjc.2016.290 PMC5117785

[16] Mackie A. Screening inequalities and what we’re doing about them. Blog. PHE Screening, https://phescreening.blog.gov.uk/2017/09/12/screening-inequalities-and-what-were-doing-about-them/ (2017, accessed 31 December 2020).

[17] Meagher S. Addressing inequalities in LGBT cancer screening coverage. Blog. PHE Screening, https://phescreening.blog.gov.uk/2019/03/15/addressing-inequalities-in-lgbt-cancer-screening-coverage/ (2019, accessed 31 December 2020).

[18] Mather S. Reducing barriers to screening for LGBT people. Blog. PHE Screening, https://phescreening.blog.gov.uk/2020/01/08/reducing-barriers-screening-lgbt/ (2020, accessed 31 December 2020).

[19] McElroy JA Wintemberg JJ Williams A. Comparison of lesbian and bisexual women to heterosexual women's screening prevalence for breast, cervical, and colorectal cancer in Missouri. LGBT Health 2015; 2: 188–192. 26790126 10.1089/lgbt.2014.0119

[20] Tanton C Soldan K Beddows S, et al. High-risk human papillomavirus (HPV) infection and cervical cancer prevention in Britain: evidence of differential uptake of interventions from a probability survey. Cancer Epidemiol Biomarkers Prev 2015; 24: 842–853. 25737331 10.1158/1055-9965.EPI-14-1333 PMC4435666

[21] Light BA Ormandy P. Lesbian gay and bisexual women in the north west: a multi-method study of cervical screening attitudes, experiences and uptake, http://usir.salford.ac.uk/id/eprint/16596 (2011, accessed 31 December 2020).

[22] Griggs J Maingi S Blinder V, et al. American society of clinical oncology position statement: strategies for reducing cancer health disparities among sexual and gender minority populations. J Clin Oncol 2017; 35: 2203–2208. 28368670 10.1200/JCO.2016.72.0441

[23] MacMillan Cancer Support, LGBT People with Cancer: The emerging picture, http://be.macmillan.org.uk/Downloads/CancerInformation/RichPicture/RP-LGBT-people-with-cancer.pdf (2016, accessed 20 May 2017).

[24] Cathcart-Rake EJ. Cancer in sexual and gender minority patients: Are we addressing their needs? Curr Oncol Rep 2018; 20: 85. 30209629 10.1007/s11912-018-0737-3

[25] Adamska L Allen N Flaig R, et al. Challenges of linking to routine healthcare records in UK biobank. Trials 2015; 16: O68.

[26] Fry A Littlejohns TJ Sudlow C, et al. Comparison of sociodemographic and health-related characteristics of UK biobank participants with those of the general population. Am J Epidemiol 2017; 186: 1026–1034. 28641372 10.1093/aje/kwx246 PMC5860371

[27] Allen N Sudlow C Downey P, et al. UK biobank: current status and what it means for epidemiology. Health Policy Technol 2012; 1: 123–126.

[28] Yousaf S Bonsall A, UK. Townsend Deprivation Scores from 2011 census data. UK Data Archive, www.statistics.digitalresources.jisc.ac.uk/dataset/2011-uk-townsend-deprivation-scores. (2017, accessed 31 December 2020).

[29] Koo S Neilson LJ Von Wagner C, et al. The NHS bowel cancer screening program: current perspectives on strategies for improvement. Risk Manag Healthc Policy 2017; 10: 177–187. 29270036 10.2147/RMHP.S109116 PMC5720037

[30] Public Health England, Promotional material: Cervical screening for lesbian and bisexual women. https://www.gov.uk/government/publications/cervical-screening-lesbian-and-bisexual-women/cervical-screening-for-lesbian-and-bisexual-women (2009, accessed 31 December 2020).

[31] Davis VJ Health L. Lesbian health. The Obstetrician & Gynaecologist 2005; 7: 98–102.

[32] Marchand GJ Sainz KM. Pap smear ransom – is it ethical to refuse to refill a patient's birth control until they come in for their annual exam? Int J Womens Health 2020; 12: 265–267. 32308500 10.2147/IJWH.S246220 PMC7153914

[33] Bailey JV Farquhar C Owen C, et al. Sexual behaviour of lesbians and bisexual women. Sex Transm Infect 2003; 79: 147–150. 12690139 10.1136/sti.79.2.147 PMC1744617

[34] Alencar Albuquerque G de Lima Garcia C da Silva QG, et al. Access to health services by lesbian, gay, bisexual, and transgender persons: systematic literature review. BMC Int Health Hum Rights 2016; 16: 2. 26769484 10.1186/s12914-015-0072-9 PMC4714514

[35] Hulbert-Williams NJ Plumpton C Flower P, et al. The cancer care experiences of gay, lesbian and bisexual patients: a secondary analysis of data from the UK cancer patient experience survey. Eur J Cancer Care 2017; 26: e12670. 10.1111/ecc.12670 28239936

[36] Brooks H Llewellyn CD Nadarzynski T, et al. Sexual orientation disclosure in health care: a systematic review. Br J Gen Pract 2018; 68: e187–e196. 29378698 10.3399/bjgp18X694841 PMC5819984

[37] Fraumeni JF Lloyd JW Smith EM, et al. Cancer mortality among nuns: role of marital status in etiology of neoplastic disease in women. J Natl Cancer Inst 1969; 42: 455–468. 5777491

[38] Nuffield Trust. How has cervical screening coverage in England changed over time?, www.nuffieldtrust.org.uk/chart/how-has-cervical-screening-coverage-in-england-changed-over-time-2 (2020, accessed 31 December 2020).

[39] Geary RS Tanton C Erens B, et al. Sexual identity, attraction and behaviour in Britain: the implications of using different dimensions of sexual orientation to estimate the size of sexual minority populations and inform public health interventions. PLoS One 2018; 13: e0189607. 29293516 10.1371/journal.pone.0189607 PMC5749676

