# Supplementary material for: Cervical screening attendance and cervical cancer risk among women who have sex with women
Source: J Med Screen. 2021 Jan 21;28(3):349–56. doi: 10.1177/0969141320987271 (PMC8366122; doi:10.1177/0969141320987271)
Supplement: sj-pdf-1-msc-10.1177_0969141320987271 - Supplemental material for Cervical screening attendance and cervical cancer risk among women who have sex with women [file sj-pdf-1-msc-10.1177_0969141320987271.pdf]

Appendix Figure 1. Identification of sexual history in UK Biobank (both women and men)

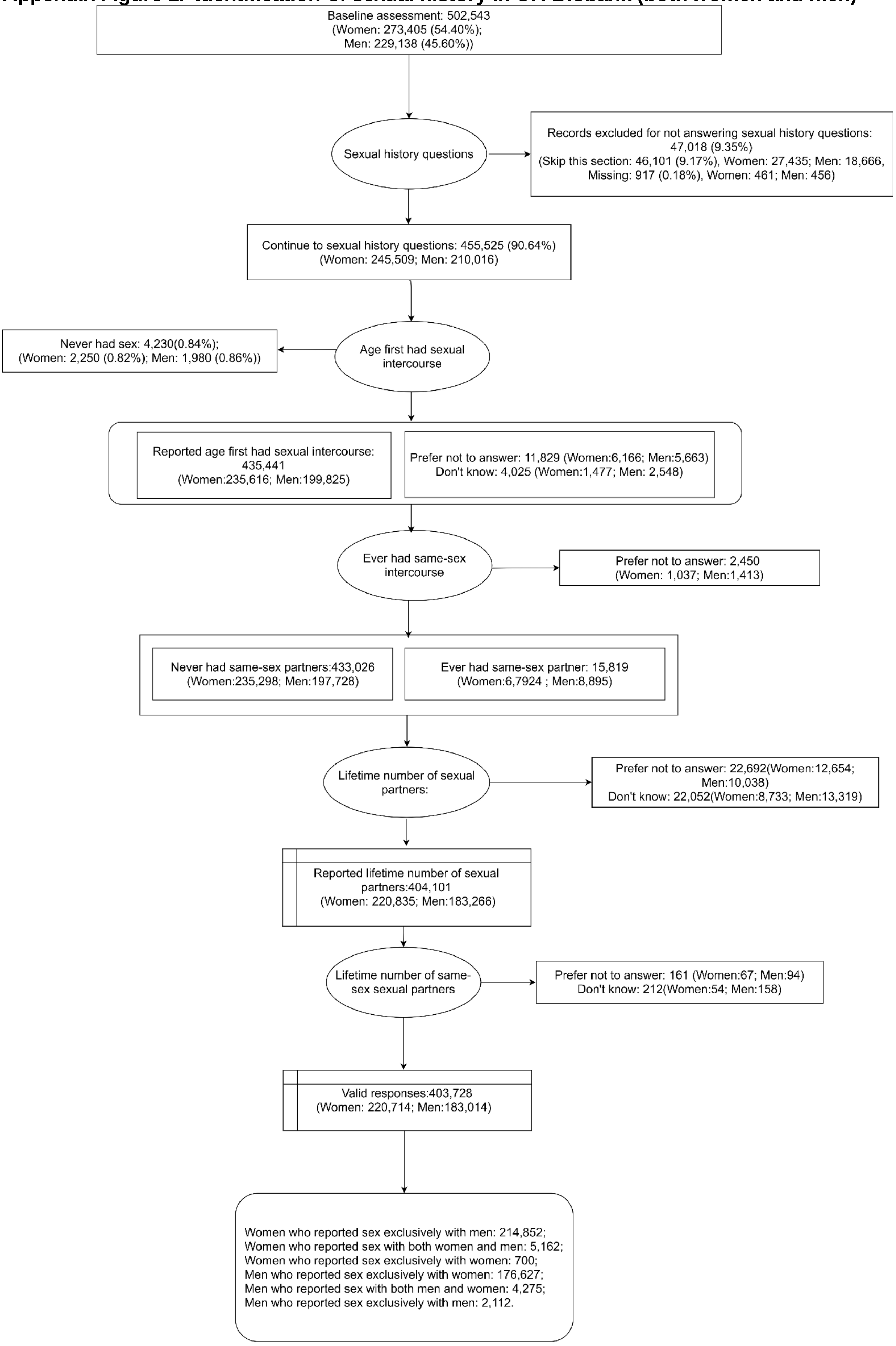

Appendix Table 1: Flow chart

	Number	Excluded (%)
Women in UK Biobank (January 2020)	273349	
Excluding women with and missing sexual history with either women or men	222818	50531 (22.7)
Excluding women with missing deprivation, ethnicity, smoking history	221227	1591 (0.7)
Excluding women with missing screening history	218674	2553 (1.2)
Final analysis sample size	218674	

Appendix Table 2. Sexual history, history of CIN3 and cervical cancer, stratified by age

	Under 45	45-49	50-54	55-59	60-64	65 and over
**Sexual history**						
No history of sex with either women or men	270 (1.2)	335 (1.1)	348 (1.0)	372 (0.9)	484 (0.9)	383 (1.1)
Sex exclusively with women (WSEW)	155 (0.7)	175 (0.6)	124 (0.4)	89 (0.2)	89 (0.2)	52 (0.1)
Sex exclusively with men (WSEM)	21550 (93.4)	28681 (94.5)	33777 (95.8)	39829 (96.9)	51370 (97.7)	35659 (98.2)
Sex with both women and men (WSWM)	1090 (4.7)	1157 (3.8)	1019 (2.9)	815 (2.0)	624 (1.2)	227 (0.6)
**Lifetime number of sexual partners**						
0	270 (1.2)	335 (1.1)	348 (1.0)	372 (0.9)	484 (0.9)	383 (1.1)
1	3614 (15.7)	6078 (20.0)	8535 (24.2)	13205 (32.1)	21848 (41.6)	18226 (50.2)
2 to 3	4693 (20.3)	7005 (23.1)	8936 (25.3)	11013 (26.8)	14477 (27.5)	9980 (27.5)
4 to 5	4576 (19.8)	5943 (19.6)	6775 (19.2)	7021 (17.1)	7621 (14.5)	4204 (11.6)
6 or more	9912 (43.0)	10987 (36.2)	10674 (30.3)	9494 (23.1)	8137 (15.5)	3528 (9.7)
**Lifetime number of same sex partners**						
0	21820 (94.6)	29016 (95.6)	34125 (96.8)	40201 (97.8)	51854 (98.6)	36042 (99.2)
1	504 (2.2)	598 (2.0)	549 (1.6)	462 (1.1)	375 (0.7)	169 (0.5)
2 to 3	393 (1.7)	392 (1.3)	322 (0.9)	249 (0.6)	208 (0.4)	63 (0.2)
4 to 5	140 (0.6)	154 (0.5)	119 (0.3)	96 (0.2)	83 (0.2)	27 (0.1)
6 or more	208 (0.9)	188 (0.6)	153 (0.4)	97 (0.2)	47 (0.1)	20 (0.1)
**Lifetime number of opposite sex partners**						
0	425 (1.8)	510 (1.7)	472 (1.3)	461 (1.1)	573 (1.1)	435 (1.2)
1	3703 (16.1)	6171 (20.3)	8615 (24.4)	13278 (32.3)	21919 (41.7)	18243 (50.2)
2 to 3	4747 (20.6)	7044 (23.2)	8973 (25.4)	11044 (26.9)	14476 (27.5)	9961 (27.4)
4 to 5	4541 (19.7)	5895 (19.4)	6744 (19.1)	6993 (17.0)	7585 (14.4)	4203 (11.6)
6 or more	9649 (41.8)	10728 (35.3)	10464 (29.7)	9329 (22.7)	8014 (15.2)	3479 (9.6)
**Any incident or prevalent CIN3**						
No	22244 (96.4)	29470 (97.1)	34298 (97.2)	40233 (97.9)	51805 (98.6)	35996 (99.1)
Yes	821 (3.6)	878 (2.9)	970 (2.8)	872 (2.1)	762 (1.4)	325 (0.9)
**Any incident or prevalent cervical cancer**						
No	22995 (99.7)	30247 (99.7)	35106 (99.5)	40940 (99.6)	52359 (99.6)	36236 (99.8)
Yes	70 (0.3)	101 (0.3)	162 (0.5)	165 (0.4)	208 (0.4)	85 (0.2)

Appendix Table 3. History of prevalent CIN3 at baseline assessment and screening history, stratified by cohort characteristics

	History of prevalent CIN3 at baseline assessment		Cervical screening (all ages)		Bowel screening (age 60+)		Breast screening (age 50+)	
	No	Yes	Never screened	Ever screened	Never screened	Ever screened	Never screened	Ever screened
**Age at baseline assessment**								
Under 45	22331 (10.4)	734 (16.7)	354 (8.9)	22711 (10.6)				
45-49	29526 (13.8)	822 (18.7)	433 (10.9)	29915 (13.9)				
50-54	34342 (16.0)	926 (21.1)	466 (11.8)	34802 (16.2)			5471 (82.4)	29797 (18.8)
55-59	40265 (18.8)	840 (19.2)	590 (14.9)	40515 (18.9)			520 (7.8)	40585 (25.6)
60-64	51824 (24.2)	743 (16.9)	993 (25.1)	51574 (24.0)	26803 (61.2)	25764 (57.2)	372 (5.6)	52195 (32.9)
65+	36000 (16.8)	321 (7.3)	1121 (28.3)	35200 (16.4)	17021 (38.8)	19300 (42.8)	280 (4.2)	36041 (22.7)
**Lifetime number of sexual partners**								
0	2189 (1.0)	3 (0.1)	1001 (25.3)	1191 (0.6)	454 (1.0)	413 (0.9)	123 (1.9)	1464 (0.9)
1	71114 (33.2)	392 (8.9)	1170 (29.6)	70336 (32.8)	20048 (45.7)	20026 (44.4)	1623 (24.4)	60191 (37.9)
2 to 3	55173 (25.7)	931 (21.2)	899 (22.7)	55205 (25.7)	12068 (27.5)	12389 (27.5)	1636 (24.6)	42770 (27.0)
4 to 5	35156 (16.4)	984 (22.4)	461 (11.7)	35679 (16.6)	5811 (13.3)	6014 (13.3)	1269 (19.1)	24352 (15.4)
6 or more	50656 (23.6)	2076 (47.3)	426 (10.8)	52306 (24.4)	5443 (12.4)	6222 (13.8)	1992 (30.0)	29841 (18.8)
**Lifetime number of same sex partners**								
0	208815 (97.4)	4243 (96.7)	3807 (96.2)	209251 (97.5)	43342 (98.9)	44554 (98.9)	6396 (96.3)	155826 (98.2)
1	2582 (1.2)	75 (1.7)	45 (1.1)	2612 (1.2)	273 (0.6)	271 (0.6)	114 (1.7)	1441 (0.9)
2 to 3	1588 (0.7)	39 (0.9)	57 (1.4)	1570 (0.7)	128 (0.3)	143 (0.3)	71 (1.1)	771 (0.5)
4 to 5	609 (0.3)	10 (0.2)	23 (0.6)	596 (0.3)	51 (0.1)	59 (0.1)	24 (0.4)	301 (0.2)
6 or more	694 (0.3)	19 (0.4)	25 (0.6)	688 (0.3)	30 (0.1)	37 (0.1)	38 (0.6)	279 (0.2)
**Lifetime number of opposite sex partners**								
0	2863 (1.3)	13 (0.3)	1073 (27.1)	1803 (0.8)	525 (1.2)	483 (1.1)	152 (2.3)	1789 (1.1)
1	71529 (33.4)	400 (9.1)	1171 (29.6)	70758 (33.0)	20082 (45.8)	20080 (44.6)	1643 (24.7)	60412 (38.1)
2 to 3	55307 (25.8)	938 (21.4)	876 (22.1)	55369 (25.8)	12064 (27.5)	12373 (27.5)	1644 (24.7)	42810 (27.0)
4 to 5	34980 (16.3)	981 (22.4)	446 (11.3)	35515 (16.5)	5805 (13.2)	5983 (13.3)	1256 (18.9)	24269 (15.3)
6 or more	49609 (23.2)	2054 (46.8)	391 (9.9)	51272 (23.9)	5348 (12.2)	6145 (13.6)	1948 (29.3)	29338 (18.5)
**Deprivation**								
Least deprived	82627 (38.6)	1373 (31.3)	1160 (29.3)	82840 (38.6)	18279 (41.7)	18070 (40.1)	2259 (34.0)	63595 (40.1)
2	45720 (21.3)	886 (20.2)	761 (19.2)	45845 (21.4)	9822 (22.4)	9851 (21.9)	1378 (20.7)	34505 (21.8)
3	31983 (14.9)	670 (15.3)	604 (15.3)	32049 (14.9)	6252 (14.3)	6619 (14.7)	993 (14.9)	23295 (14.7)
4	27841 (13.0)	679 (15.5)	632 (16.0)	27888 (13.0)	5030 (11.5)	5607 (12.4)	958 (14.4)	19628 (12.4)
Most deprived	26117 (12.2)	778 (17.7)	800 (20.2)	26095 (12.2)	4441 (10.1)	4917 (10.9)	1055 (15.9)	17595 (11.1)
**Ethnicity**								
White	205732 (96)	4281 (97.6)	3565 (90.1)	206448 (96.1)	43014 (98.2)	44170 (98.0)	6194 (93.2)	154187 (97.2)
Mixed	1073 (0.5)	23 (0.5)	27 (0.7)	1069 (0.5)	103 (0.2)	121 (0.3)	49 (0.7)	535 (0.3)
Asian	2487 (1.2)	15 (0.3)	142 (3.6)	2360 (1.1)	285 (0.7)	254 (0.6)	147 (2.2)	1290 (0.8)
Black	2853 (1.3)	37 (0.8)	118 (3.0)	2772 (1.3)	229 (0.5)	265 (0.6)	153 (2.3)	1389 (0.9)
Other	2143 (1.0)	30 (0.7)	105 (2.7)	2068 (1.0)	193 (0.4)	254 (0.6)	100 (1.5)	1217 (0.8)
**Smoking history**								
Never	130054 (60.7)	1934 (44.1)	2688 (67.9)	129300 (60.2)	25491 (58.2)	25775 (57.2)	4149 (62.5)	93533 (59)
Former	66550 (31.1)	1660 (37.8)	910 (23.0)	67300 (31.3)	15236 (34.8)	16733 (37.1)	1738 (26.2)	53363 (33.6)
Current	17684 (8.3)	792 (18.1)	359 (9.1)	18117 (8.4)	3097 (7.1)	2556 (5.7)	756 (11.4)	11722 (7.4)
**Year of baseline assessment**								
2006	1631 (0.8)	37 (0.8)	36 (0.9)	1632 (0.8)	395 (0.9)	141 (0.3)	54 (0.8)	1118 (0.7)
2007	21285 (9.9)	401 (9.1)	477 (12.1)	21209 (9.9)	5654 (12.9)	2281 (5.1)	758 (11.4)	15039 (9.5)
2008	80505 (37.6)	1809 (41.2)	1372 (34.7)	80942 (37.7)	21413 (48.9)	11667 (25.9)	2482 (37.4)	59793 (37.7)
2009	73751 (34.4)	1477 (33.7)	1381 (34.9)	73847 (34.4)	12355 (28.2)	18859 (41.8)	2146 (32.3)	54993 (34.7)
2010	37116 (17.3)	662 (15.1)	691 (17.5)	37087 (17.3)	4007 (9.1)	12116 (26.9)	1203 (18.1)	27675 (17.4)

Appendix Table 3 – alternative format. History of prevalent CIN3 at baseline assessment and screening history, stratified by cohort characteristics (percentages for each group)

	History of prevalent CIN3 at baseline assessment		Cervical screening (all ages)		Bowel screening (age 60+)		Breast screening (age 50+)	
	No	Yes	Never screened	Ever screened	Never screened	Ever screened	Never screened	Ever screened
**Age at baseline assessment**								
Under 45	96.8	3.2	1.5	98.5				
45-49	97.3	2.7	1.4	98.6				
50-54	97.4	2.6	1.3	98.7			15.5	84.5
55-59	98.0	2.0	1.4	98.6			1.3	98.7
60-64	98.6	1.4	1.9	98.1	51.0	49.0	0.7	99.3
65+	99.1	0.9	3.1	96.9	46.9	53.1	0.8	99.2
**Lifetime number of sexual partners**								
0	99.9	0.1	45.7	54.3	52.4	47.6	7.8	92.2
1	99.5	0.5	1.6	98.4	50.0	50.0	2.6	97.4
2 to 3	98.3	1.7	1.6	98.4	49.3	50.7	3.7	96.3
4 to 5	97.3	2.7	1.3	98.7	49.1	50.9	5.0	95.0
6 or more	96.1	3.9	0.8	99.2	46.7	53.3	6.3	93.7
**Lifetime number of same sex partners**								
0	98.0	2.0	1.8	98.2	49.3	50.7	3.9	96.1
1	97.2	2.8	1.7	98.3	50.2	49.8	7.3	92.7
2 to 3	97.6	2.4	3.5	96.5	47.2	52.8	8.4	91.6
4 to 5	98.4	1.6	3.7	96.3	46.4	53.6	7.4	92.6
6 or more	97.3	2.7	3.5	96.5	44.8	55.2	12.0	88.0
**Lifetime number of opposite sex partners**								
0	99.5	0.5	37.3	62.7	52.1	47.9	7.8	92.2
1	99.4	0.6	1.6	98.4	50.0	50.0	2.6	97.4
2 to 3	98.3	1.7	1.6	98.4	49.4	50.6	3.7	96.3
4 to 5	97.3	2.7	1.2	98.8	49.2	50.8	4.9	95.1
6 or more	96.0	4.0	0.8	99.2	46.5	53.5	6.2	93.8
**Deprivation**								
Least deprived	98.4	1.6	1.4	98.6	50.3	49.7	3.4	96.6
2	98.1	1.9	1.6	98.4	49.9	50.1	3.8	96.2
3	97.9	2.1	1.8	98.2	48.6	51.4	4.1	95.9
4	97.6	2.4	2.2	97.8	47.3	52.7	4.7	95.3
Most deprived	97.1	2.9	3.0	97.0	47.5	52.5	5.7	94.3
**Ethnicity**								
White	98.0	2.0	1.7	98.3	49.3	50.7	3.9	96.1
Mixed	97.9	2.1	2.5	97.5	46.0	54.0	8.4	91.6
Asian	99.4	0.6	5.7	94.3	52.9	47.1	10.2	89.8
Black	98.7	1.3	4.1	95.9	46.4	53.6	9.9	90.1
Other	98.6	1.4	4.8	95.2	43.2	56.8	7.6	92.4
**Smoking history**								
Never	98.5	1.5	2.0	98.0	49.7	50.3	4.2	95.8
Former	97.6	2.4	1.3	98.7	47.7	52.3	3.2	96.8
Current	95.7	4.3	1.9	98.1	54.8	45.2	6.1	93.9
**Year of baseline assessment**								
2006	97.8	2.2	2.2	97.8	73.7	26.3	4.6	95.4
2007	98.2	1.8	2.2	97.8	71.3	28.7	4.8	95.2
2008	97.8	2.2	1.7	98.3	64.7	35.3	4.0	96.0
2009	98.0	2.0	1.8	98.2	39.6	60.4	3.8	96.2
2010	98.2	1.8	1.8	98.2	24.9	75.1	4.2	95.8
