# Supplementary material for: Cervical screening attendance and cervical cancer risk among women who have sex with women
Source: J Med Screen. 2021 Jan 21;28(3):349–56. doi: 10.1177/0969141320987271 (PMC8366122; doi:10.1177/0969141320987271)
Supplement: sj-pdf-2-msc-10.1177_0969141320987271 - Supplemental material for Cervical screening attendance and cervical cancer risk among women who have sex with women [file sj-pdf-2-msc-10.1177_0969141320987271.pdf]

JOURNAL OF MEDICAL SCREENING

LICENCE FOR PUBLICATION

The *Journal of Medical Screening* owned by the Medical Screening Society and published by SAGE requires the author(s) of a contribution to grant an exclusive licence to help ensure international protection against infringement of copyright, in particular unauthorised photocopying, digital distribution, and other use by third parties, and so that it can handle requests from third parties to reproduce contributions or parts of contributions. If copyright is held by your employer please obtain the employer's express authority to grant an exclusive licence. If you are a government employee please obtain express authority to grant a non-exclusive licence to publish and exploit subsidiary rights.

If the contributions are not published in either the print or electronic versions of the *Journal of Medical Screening* within 12 months of acceptance (or as otherwise agreed) this agreement shall automatically terminate and all rights shall revert to the copyright owner.

In consideration of the publication in the *Journal of Medical Screening* of my/our contribution:

JMS Reference Number: JMS-20-165.R1

Title: Cervical screening attendance and cervical cancer risk among women who have sex with women

All Authors: Catherine L Saunders, Efthalia Massou, Jo Waller, Catherine Meads, Laura AV Marlow,

Juliet A Usher-Smith

Corresponding author name & address: Dr Catherine Saunders, The Primary Care Unit
Department of Public Health and Primary Care, University of Cambridge, School of Clinical Medicine, Box 113 Cambridge Biomedical Campus, Cambridge, CB2 0SR

I/we hereby grant to the Medical Screening Society for the full period of copyright including any renewals or extensions throughout the world and in all languages an exclusive licence to publish the above contribution or permit others to do so in print editions and in digital formats including online and network editions of the journal and in other derivative or collective works and to exploit subsidiary rights in the contributions, including database rights.

I/we confirm that I am/we are the sole author(s) of the contribution which is my/our original work. It has not been previously published in whole or substantial part. I am/we are the copyright owner of the contribution or am/are expressly authorised by the copyright owner to grant this licence. I/we confirm that I/we have used our reasonable endeavours to ensure that nothing of a technical nature in this paper is materially inaccurate, that it is in no way whatever a violation of any existing copyright or a breach of any existing agreement, and that it contains nothing defamatory or libellous.

In return for the grant of the exclusive licence, the contributor(s) (or, if copyright is vested in the contributor(s)'s employer, the contributor(s)'s employer) shall have the following rights:

- The right to use and permit colleagues to use print or electronic "preprints"(drafts) of the unpublished contribution(s). Such preprints may be posted as electronic files on the contributor's own web site for personal or professional use, or the contributor's internal university or corporate network or secure external

Please sign next page

network at the contributor’s institution, but not for commercial sale or other commercial exploitation or for any other systematic external distribution by a third party (e.g. list serve or database connected to a public access server). Prior to circulating or otherwise making available this contribution, the contributor(s) must include the following notice on the preprint: “ This is a preprint of an article accepted for publication by *Journal of Medical Screening* and may not save under the fair dealing of the provision of the Copyright Designs and Patents Act (1988) be reproduced without the consent of the Medical Screening Society”

After publication of the contribution, the preprint must be replaced with a reference or electronic link to the published version, or if the preprint remains, the notice should be amended to read as follows: “ This is a preprint of an article published in the *Journal of Medical Screening* **(**digital and paper citation) and may not save under fair dealing provisions be reproduced without the consent of the Medical Screening Society.

•The right to reproduce a reasonable number of copies of the contribution, by photocopying or downloading from the on line version of *Journal of Medical Screening*, for personal or professional (non-commercial) use. This use includes the contributor(s)’s own teaching purposes.

•The right to post, with the necessary acknowledgement and link to the *Journal of Medical Screening* website, the contribution on the contributor(s)’s own, or the contributor(s)’s institution’s, website

•The right to publish with the necessary acknowledgement all or part of the material from the published contribution in a book written or edited by the contributor(s). This does not apply to multiple contributions in the same journal, for which permission must be sought.

•The right to use selected figures and tables and selected text (up to 250 words) from the contribution for incorporation within another work written by the contributor that is made part of an edited work published in print or digital format by a third party.

•The right to include the contribution in a compilation for classroom use (course packs) to be distributed free of charge to students at the contributor’s institution or to be stored in digital format in datarooms for access by students as part of their course work and for in house training programmes of the contributor(s)’s employer. This does not apply if any charge is made for the compilation (other than photocopying costs) or the training programme.

•The right to receive a royalty of 10% of any net receipts less sales commission on orders in excess of £1000 received by the publisher from any reprint sales or rights granted to a third party (such as translation rights). The contributor must complete the Royalty assignment form to assign the right to receive this royalty to a single named individual or institution.

Copyright: exclusive licence

The author who signs this agreement certifies that he/she is authorized to sign on behalf of him/herself, and in the case of a multi-authored contribution on behalf of all other authors of the contribution. NB: Any co-author(s) understand that they each have the option of signing and returning a separate copy of this agreement.

Contributor signature:	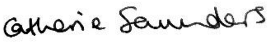
Contributor name (please **print** clearly):	Catherine Saunders
Date:	18th December 2020

PLEASE RETURN AS SOON AS POSSIBLE SO THAT PUBLICATION IS NOT DELAYED to:
Ms Karuna Rana, Production Editor
Karuna.Rana@sagepub.in

ONLINE ACCESS OPTIONS

All Journal papers are available free online two years after publication; however, the Journal is now offering authors of accepted manuscripts two options for online access to their papers. Under the existing arrangement (option 1), all readers other than Journal subscribers must pay to download the paper. Under the new alternative (option 2), authors may pay a fee to enable their paper to be freely accessible online. The fee is $3000 (US) (approx £1850). Would you please indicate below which of these two options you would prefer.

Option 1 (no payment required from authors – readers pay to access for two years) [X]

Signature of corresponding author (please print name alongside):

Catherine Saunders Catherine Saunders
